# Mobile Health in Remote Patient Monitoring for Chronic Diseases: Principles, Trends, and Challenges

**DOI:** 10.3390/diagnostics11040607

**Published:** 2021-03-29

**Authors:** Nora El-Rashidy, Shaker El-Sappagh, S. M. Riazul Islam, Hazem M. El-Bakry, Samir Abdelrazek

**Affiliations:** 1 Machine Learning and Information Retrieval Department, Faculty of Artificial Intelligence, Kafrelsheiksh University, Kafrelsheiksh 13518, Egypt; noura.alrashidy@ai.kfs.edu.eg; 2 Centro Singular de Investigación en Tecnoloxías Intelixentes (CiTIUS), Universidade de Santiago de Compostela, 15782 Santiago de Compostela, Spain; 3 Information Systems Department, Faculty of Computers and Artificial Intelligence, Benha University, Banha 13518, Egypt; 4 Department of Computer Science and Engineering, Sejong University, Seoul 05006, Korea; 5 Information Systems Department, Faculty of Computers and Information, Mansoura University, Mansoura 13518, Egypt; elbakry@mans.edu.eg (H.M.E.-B.); samir.abdelrazek@mans.edu.eg (S.A.)

**Keywords:** electronic health, electronic health record, clinical-decision support system, AI, remote patient monitoring, cloud computing, internet of things, wireless body area network

## Abstract

Chronic diseases are becoming more widespread. Treatment and monitoring of these diseases require going to hospitals frequently, which increases the burdens of hospitals and patients. Presently, advancements in wearable sensors and communication protocol contribute to enriching the healthcare system in a way that will reshape healthcare services shortly. Remote patient monitoring (RPM) is the foremost of these advancements. RPM systems are based on the collection of patient vital signs extracted using invasive and noninvasive techniques, then sending them in real-time to physicians. These data may help physicians in taking the right decision at the right time. The main objective of this paper is to outline research directions on remote patient monitoring, explain the role of AI in building RPM systems, make an overview of the state of the art of RPM, its advantages, its challenges, and its probable future directions. For studying the literature, five databases have been chosen (i.e., science direct, IEEE-Explore, Springer, PubMed, and science.gov). We followed the (Preferred Reporting Items for Systematic Reviews and Meta-Analyses) PRISMA, which is a standard methodology for systematic reviews and meta-analyses. A total of 56 articles are reviewed based on the combination of a set of selected search terms including RPM, data mining, clinical decision support system, electronic health record, cloud computing, internet of things, and wireless body area network. The result of this study approved the effectiveness of RPM in improving healthcare delivery, increase diagnosis speed, and reduce costs. To this end, we also present the chronic disease monitoring system as a case study to provide enhanced solutions for RPMs.

## 1. Introduction

Chronic diseases are physical or mental conditions such as hypertension, diabetes, cardiovascular, obesity, stroke, etc. These diseases constitute the bulk of human health risks, responsible for more than two-thirds of all deaths worldwide [1]. The incidence of chronic disease has increased along with population growth, hospital capacity is insufficient to accommodate all patients. Besides, chronic diseases require special home care to fulfill patients’ needs or administer therapy programs. Besides, most caregivers and families do not have the time or skills, as a result, patients’ quality of life is always at risk [2]. 

Developing e-health systems (e.g., remote patient monitoring (RPM), electronic health record (EHR) systems, mobile health (m-health), telemedicine, e-visits, e-consultations, etc.) is an increasing need. Such systems are used for continuous monitoring, diagnosis, prediction, and treatment. Consequently, they contribute to reducing healthcare costs and allow patients to perform their daily activities while their vital signs are fully monitored [3]. Besides, these systems allow physicians to follow up with patients at any time, not just when patients are physically present at a hospital. Patient monitoring (PM) systems work to empower patients with knowledge about their symptoms and treatments, helping them live independently which helps to increase their quality of life [4]. On the other hand, PM systems are important in hospitals; for example, they can be used to rank patients based on their conditions, allowing hospitals to prioritize critical patient care. 

The widespread use of smart mobile devices has significantly affected the number of patients using healthcare systems. The number of patients using mobile devices has increased from 35,000 in 2013 to 7 million in 2018 [5]. Therefore, RPMs have a significant impact on patients with various domains. In [6], the authors provide a survey that studies the effect of RPM systems on a patient with spinal cord injury (SCI), it concluded that PM systems were influential and promising in the control or prevention of complication for SCI patients and could be considered in therapy planning. Others such as [7] provide a literature review for RPM. It concluded by highlighting the importance of RPM as it allowed physicians to monitor several patients in parallel. The authors of [8] provide a Systematic review for RPM, which concentrated on the role of PMs with different diseases. Others like [9] concentrate on the most recent application developed for PMs.

The Internet of things (IoT) is a new technology used to make all objects smart. The IoT has a high impact on various domains [10], among which the medical domain is considered the most attractive. IoT has the potential to automatically connect sensors, devices, and patients without human intervention through remote monitoring systems. A wireless body area network (WBAN) is an IoT subdomain. It is a wireless sensor network that connects wearable devices, called sensors, on a patient’s body, to the network, allowing remote monitoring for the patient’s vital signs. In the medical domain, a WBAN consists of a small network of sensors, such as a pulse oximeter, gyroscopes, a spirometer, a global positioning system (GPS), and electrooculography (ECO) [11,12],

Decision support systems (DSS) have a major role in both physician and patient. They not only help physicians in diagnosis and treatment but also improve healthcare remotely, which affects the patients’ quality life [13,14]. However, the quality of CDSS is highly dependent on the quality of clinical data. If the collected data is imprecise, CDSS will result in wrong decisions. In this case, it will not be applicable in the medical domain and acceptable by physicians. CDSS is mainly based on a knowledge base. To improve its knowledge, the semantic web is an efficient solution for knowledge sharing and representation. Ontology is one of the pillars of the semantic web. It is defined as “an adapted technology for knowledge representation” [15]. It acts like a dictionary for a specific domain that defines objects, properties, and the relationship between these objects [16,17]. In this regard, this paper contributes by:

(a) Studying 56 papers in the period of (2015–2019) that cover several features related to RPMS, including IoT, WBAN, cloud computing, fog computing, and CDSS.
(b) Providing a comprehensive survey that summarizes the state of the art of RPM systems, tools, technologies, recent applications, and techniques.
(c) Highlighting all the steps in building efficient and effective RPMs, in addition to the challenges and future directions at each stage.
(d) Discussing the importance of artificial intelligence (AI) in building medically intuitive monitoring systems.
(e) Providing a case study of remote patient monitoring for chronic diseases patients that tries to cover several limitations of the state-of-the-art architectures.

The significance of our study is to highlight the importance of RPMs as a technological innovation in the healthcare sector and show the progression of the related technologies. The paper is organized as the following: Section 2 introduces the search strategy. The literature review for all components is discussed in Section 3. Section 4 summarizes disease-specific remote patient monitoring systems. In Section 5, the paper provides an overview of the role of AI in building efficient patient monitoring systems. While Section 6 provides a case study for a chronic disease monitor system, Section 7 summarizes the current RPMs challenges and provides future directions. The final section concludes this review. For the convenience of readers, the most commonly used abbreviations are listed at the end of the manuscript.

## 2. Materials and Methods

Our research embraces the (Preferred Reporting Items for Systematic Reviews and Meta-Analyses) PRISMA methodology that includes all search details such as (research questions, literature review, and strategy details). 

In this research we concentrate on the following questions:

What is the main role of RPMs in the healthcare sector?

To what extent do patients and their relatives become confident on PMs?

What is the role of ontology in unifying electronic medical records across all the hospitals?

What is the importance of RPMs from a physician’s perspective?

### 2.1. Selection Criteria 

**Figure 1:**
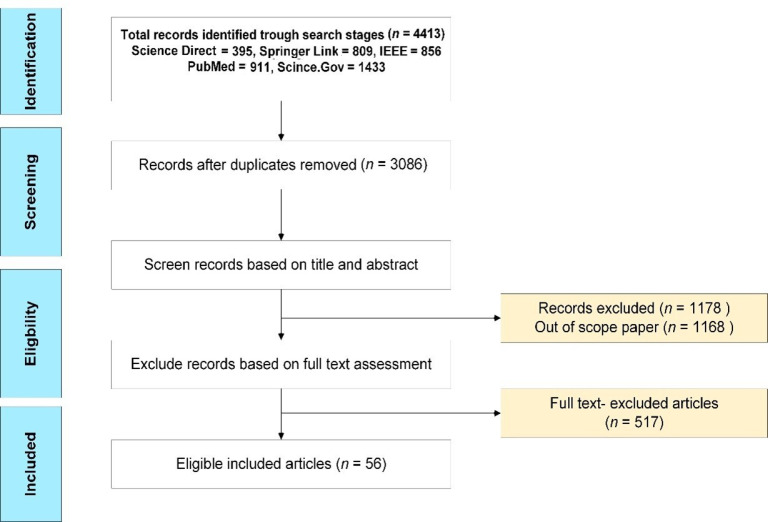
Steps used to select articles.

First, we concentrate on five main search engines (IEEE, Springer, PubMed, Scince.Gov, and Science Direct); second, selection-based RPMs’ relevancy degree to the following keywords (Clinical decision support systems, internet of things, wireless body area network, cloud computing). Third, some papers were excluded due to duplications. Finally, all literature was screened, and we finally ended up with 52 articles. Figure 1 shows the selection and evaluation process. The paper concentrated on studying the literature of RPMs in the last five years (i.e., from 2015 to 2019). We decided to exclude 2020 from the survey because the study was finished before the end of this year. Table 1 shows the keywords that were used to obtain eligible articles and the aggregated articles according to each keyword.

### 2.2. Results Statistical Analysis 

**Figure 2:**
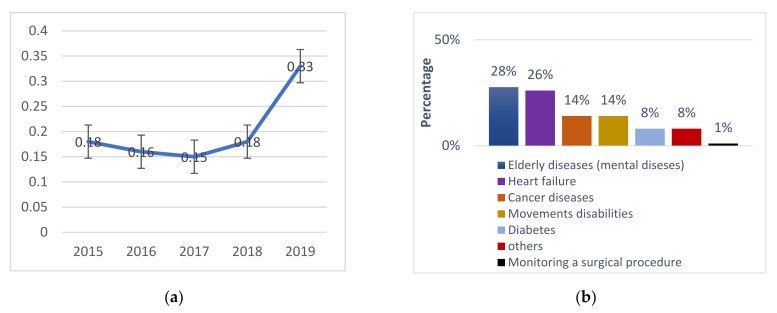
(**a**) Number of articles per year; (**b**) Distribution of RPMs according to diseases.

This section provides a statistical analysis of the search results from various points of view (different domains, differences in each year). Figure 2a shows the distribution of the published articles during the period of our study (2014 to 2019). As shown, the number of studies rapidly increases from 18% in 2014 to 33% in 2019. It indicates the growth in the research related to RPMs. Figure 2b shows the distribution of the gathered articles according to the paper’s application diseases. According to these distributions, we can notice that PMs for the elderly take the largest percentage (28%) followed by PMs for heart failure (26%). Other diseases such as cancer, diabetes, and post-surgical take proximate percentage ranges from 8 to 14%.

## 3. Main Components of the RPM System

According to the literature, standard RPMs are intended to continuously capture many clinical data from patients and allow physicians to be continuously monitored using various internal and external sensors. The main steps of building PMs can be summarized as follows, (1) Data acquisition: Vital signs are continuously monitored using invasive and no invasive techniques. This step is used to extract vital signs such as (EEG, ECG, blood pressure, heart rate, etc.). In addition to these vital signs, devices are used to gather other context variables such as (room temperature, pressure, etc.). (2) Data transmitting and storing: All data are aggregated and transmitted to the cloud side for analyzing, sorting, and processing. Cloud data could be accessed from different sources include (laboratory, ambulance, clinics, pharmacy, etc.). (3) Backend systems: All data are analyzed, then used to help physicians with real-time decisions about patient status. The following points detail the advantages of RPMS in the medical domain.

**Figure 3:**
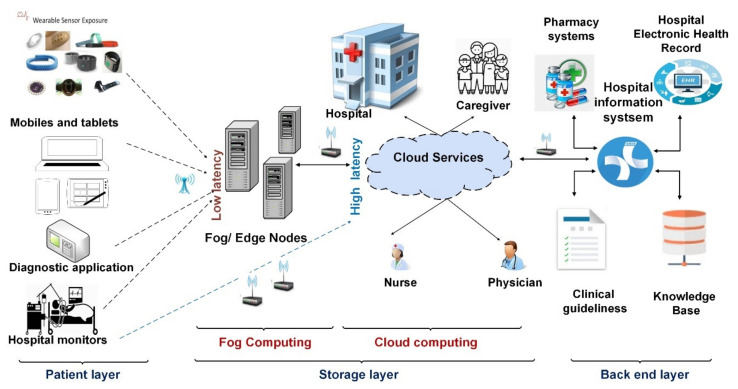
The general architecture of RPMs.

- Provide patient assurance: RPMs could provide (24/7) care at home through wearable sensors, which are used to frequently measure patient vital signs, provide a real-time recommendation based on patient status.
- Increase patient awareness and responsibility: the continuous collecting of patient data increase patient awareness about his/her health status.
- Provision of low-cost solutions: depending on RPMs decreases the cost of hospitalization and admissions, consequently, saving on the total cost of healthcare services. Figure 3 shows the general form of the patient monitoring system.

### 3.1. Data Acquisition

Sensors play an essential role in most RPMs. They are considered a bridge between the patient and the physical world [18]. RPMs used various sensors to aggregate patient’s vital signs and health data such as (EEG, ECG, heart rate, etc.), in addition to context such as (room temperature, room oxygen level, etc.). Internet of things (IoT) devices are used to transmit data among several networks [19,20]. This provides human-to-human, thing-to-thing interactions through a set of sensors and devices. A wireless body area network is a set of wearable sensors that are usually attached to a patient’s body using invasive and noninvasive techniques. WBAN sensor nodes are classified into two main types as follows:

- Implanted sensors: sensors that are implanted inside the patient’s body (under the patient’s skin).
- External sensors: sensors that attached directly to the patient’s skin or separated with about (2–5) CM.

#### WBAN Challenges in RMS

Despite the advantages of WBAN sensors in RPMs. It still has several challenges and limitations which affect efficiency and reliability, these challenges could be listed as follows: 

Transmission protocols: Most healthcare systems need to transmit data across local and global networks using wireless standards such as ZigBee, Lora, Bluetooth [21]. Each wireless standard has limitations related to power, energy, and range of transmission. For limited area applications, ZigBee is considered suitable [20]. Table 2 includes a summarization of transmission protocols. 

Data privacy and security: Most patients using RPMs are usually concerned about their health data, therefore various studies are concerned with data privacy and security [22,23]. For example, in [24], the authors proposed a management schema based on elliptic curve cryptography (ECC). ECC is a security schema that divides into three steps. (1) Identification of data skin and consumers, (2) Registration confirms the identification step and creates a secret channel, (3) Verify the communication between skin and consumer. The authors concluded that this schema enhances the reliability of the system. Table 3 showed a description of the collected articles according to various factors include (Diseases, collected data, the sensor used and the transmission protocol). The authors of [25], provide a comprehensive survey about security and privacy among WS devices. 

Interoperability and integration: Health data are usually big and heterogeneous; therefore, interoperability is a critical issue. In PMs, interoperability works on two main sides. (1) Sensor interoperability: integrated sensors and remote devices in an integrated sensing system. (2) Data interoperability: combine data from heterogeneous resources (i.e., XML, CSV, SQL, etc.). provide surveys of WSN interoperability [20,36,37].

Interference: WBAN system interference is divided into two main types. (1) Intra- interference usually occurs in a single WBAN. Various studies use flexible time division multiple access (TDMA) as the most suitable that decreases interference and decreases power consuming. In [25], the author details more interference techniques in WBAN. Table 3 includes a summarization of some selected studies according to the used sensors, collected data, and the transmission protocols.

### 3.2. Storage Server

#### 3.2.1. Cloud Computing

Cloud computing in conjunction with a WSN enables promising monitoring systems that can enhance the quality of service (QoS) [38,39]. The combination offers physicians the ability to monitor all patient data sensed with biosensors regardless of the type of patient data, it contributes to reducing the burden of hospitals and clinics. For example, a cloud system for knee rehabilitation after surgery (App. A) was provided in [40], which monitored falls in elderly patients by providing a continuous monitoring system and real-time alerts. A hospital sends a request to the patient to start the monitoring process; then, a hospital doctor monitors and makes decisions based on the collected patient data. In this system, the cloud side acts as a bridge between the patients and the hospital. Another monitoring system was developed and connected to intelligent ambulance service in [41]. The idea behind this system was to link health monitoring systems with traffic control systems through the cloud, thus helping ambulances reach hospitals in the minimum amount of time, which can save patients’ lives. In [42], the authors propose a healthcare system that could access patients’ health status and predict risks. The system integrated healthcare clouds with WSNs through smartphones. Smartphone apps provided real-time updates concerning a patient’s health status to healthcare professionals via the cloud. The system also provided a filter system that compared a patient’s vital signs with normal readings saved in a lookup table. When a patient’s condition was found to be abnormal, the system sent an SMS to their doctors automatically with included patient health status and a link to the patient’s medical record saved on the cloud. In [43], the authors developed a cloud-based mobile system (CMS) for chronic diseases. This system was divided into three parts. (1) Cloud backend service acted as a storage server that gathered patient data through HTTP calls connected directly to PubNub. (2) A mobile application that performed data analysis and raised alerts of risks. (3) Mobile applications enhanced communications between the patients and his/her relatives or caregivers, authors used the impact on the family-scale (IFS) to measure the effectiveness of this system and to improve the quality of life for both patients and caregivers. Other than this, in [44], the authors developed a virtual cloud care (VCC) project to monitor elderly people. This project was based on a cloud system that stored patient sensor data in a cloud and developed logical processes to assess patient data and generate alarms. In addition to service-oriented architecture (SOA) that was utilized for standardization, in [45], authors used cloud services to build a gerontology and geriatrics healthcare system (GGHCS). The main goal of this system was to include different functions, including data querying, online diagnosis, and patient data uploading. This system worked on two sides. The server-side used a web page deployed on a cloud, while the client-side used intelligent terminals (smartphones and medical devices). Any patient can log into a web page and perform a heart-rate query and visualize heart rate curves to display heart status for the specific period range. In other words, it combined rich Internet technology with a web client to enhance analyzing and accessing medical data. In [46], the authors provided a monitoring system for chronic diseases (VISIGNET) that constantly monitored patient vital signs and reduced patient visits to the hospital or physician. This system gathered all the vital signs sensed by biosensors and automatically uploaded them to a cloud service (Xively), permitting physicians and other caregivers to remotely monitor patients in real-time. The same idea is in [47], where the authors provided a monitoring system for heart diseases. This system provided real-time monitoring and predicted future risk over the next 12 months using data mining techniques. Other researchers used cloud computing to develop teleconsultations and video conferencing systems [48,49].

Cloud computing also helps to provide uniform EHRs between hospitals [50], enhance issues related to interoperability, maintainability, scalability, portability, and so on. In [51], the authors proposed a cloud-based EHR system (CHISTAR) to address the problems faced by traditional EHR systems. This system can easily handle massive amounts of patient data from different data sources (e.g., file servers and relational databases). Ultimately, cloud computing helps to reduce costs and improve efficiency and effectiveness while developing healthcare systems, but to take full advantage of it, several challenges and limitations should be handled.

Cloud computing allows developers of cloud-based software to be away from operational and hardware concerns [52]. Therefore, most developers relied on platform-as-a-service (PaaS) and software-as-a-service (SaaS) to implement their software systems. Following a durable trend of miniaturization and commoditization of software services, we see a high concern with executing functions without putting much management burdens on the developer [53,54,55]. This architecture is associated with terms such as function-as-a-service (FaaS), which addressed various needs that were not handled by PaaS and SaaS. A comprehensive survey about FaaS is available in [56].

Limitations and challenges of cloud computing in PMs are confined to the power and time consumption, privacy, and security of the transmitted data. Several systems have been proposed to address these issues. First, some studies have focused on increasing the transmission speed by providing routes with low end-to-end delay, as in [57], a cloud health monitoring system (CHMS) was proposed to track non-hospitalization patients and improve QoS. The authors in this work try to address many challenges. First, it overcomes the delay in the delivery time of urgent data. They classified patient data as urgent and non-urgent at different frequencies, the urgent data was immediately transmitted to the medical staff, while lower-frequency data was aggregated and transmitted at a low frequency. Second, they worked on the mutual interference problem by using a dynamic channel policy that distributed WBANs to the available channel. This system also switched and selected the available channel using the master node concept. Another system has been proposed to schedule and prioritize the transmission of vital signs over multiple channels based on the patient’s current status [58]. In this system [59], The authors [60] proposed a system that suggests running some parts of the application components in parallel which helps save batteries and avoids draining too quickly. 

Regarding privacy, security, and accuracy of the transmitted data. Many solutions have been proposed to address these problems. Authors [61] provided system to handle the issues of security and privacy of transmitted data in system works from two aspects. First, it works on the secure interconnection between sensors (WBAN) and the cloud using multi-biometric key generation; second, it responds dynamically to simultaneous changes in any of patients, such as their locations. Researchers have proposed several solutions to this challenge using virtual Machines and parallel autoscaling mechanisms. Despite many attempts to find solutions to the challenges related to the use of the cloud in RPMs, many of these did not reach an acceptable result. For example, in rea-time monitoring systems, the delay result from transmitting data to the cloud and back is not acceptable. From the other hand, the continuous transmitting to the cloud server will cause high battery consumption. Relative to this context, many researchers in RPMs use the concept of fog computing which will discussed in the following subsection. 

#### 3.2.2. Fog Computing in RPMs

**Figure 4:**
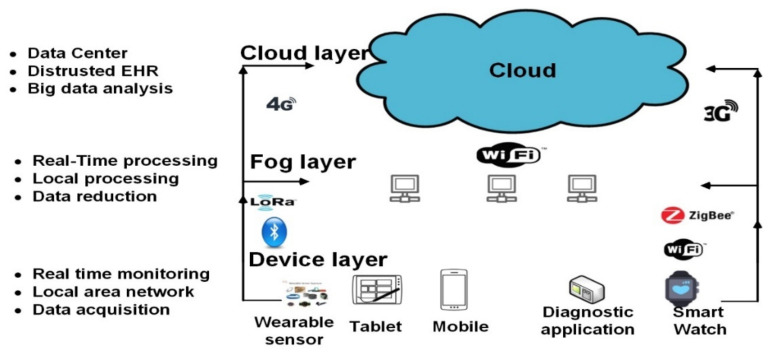
Fog computing layers in RPMs.

In 2012, cisco innovated the term fog computing [62]. Fog computing allows In 2012 cisco innovated the term of fog computing [62]. Fog computing allows applications to run on the edge of the network rather than work rather than on cloud [63]. Fog can be described as “a lightweight cloud that provide many facilities at the proximity of user’s smart device”. Fog is not a subtitle of the cloud, but it a powerful supplementary, it provides processing at the fog node, while still offering the possibility to directly interact with the cloud. The point of using fog computing is to transfer the processing of some sensitive application to the edge (near the end device), while others cane done over the cloud. Problems related to location awareness, reliability, latency and many other challenges are resolved by fog computing [64]. The fog computing in PM systems in a new concept in this domain. It provides many advantages over cloud that could summarized in the following points: (1) In fog computing, data processed and analyzed locally instead of sending it to the cloud, this led to less amount of bandwidth consuming, decrease the overall cost [65]. (2) Processing data locally will decrease the time-latency during transmission which help to avoid problems especially for time-sensitive application (e.g. real-time monitoring, self-driving car, etc.). (3) Providing better privacy to users, as patient’s data can be analyzed locally instead of sending it to the cloud (4) Deploying fog servers in PMs decrease the required bandwidth for transmission, providing real-time data to doctors without the need of internet connection [66]. (5) Utilizing fog computing will not only help people to get ease on their basic health monitoring, but will also help those countries where there is less doctor to patient ratio [63]. (6) Utilizing fog nodes provides additional advantages such as save power consumption while continuous transmitting to cloud servers [67]. Fog IoT systems divided into three main layers include (device layer, fog layer and cloud layer) instead of (device layer, cloud layer) in cloud computing systems. Table 4 show the differences between fog computing and cloud computing. In [68] authors provide a tutorial that discuss the differences between edge computing, fog computing and cloud computing, in addition to the advantages of using fog computing. Others survey [63,69] have been discussed the role of fog computing in various domains. Figure 4 show the basic of fog computing model for PMs.

In [70] authors provide smart health monitoring system based on fog nodes. In this system, edge users are equipped with various kinds of wearable sensors and devices used to aggregate medical measurements. they used LoRaWAN to send aggregated measurements directly to the closer fog nodes or health centers. Authors use LoRAWAN as it could transmit across one ten kilometers even if there is no internet connection. This will not only help patients with continuous monitoring regardless of internet connection but also decrease the overall bandwidth, the battery consumption. Others in [71] provide low-cost monitoring system of ECG and temperature. Authors use sensor node to collect data and wireless transmit to the nearest gateway accessed by caregivers. It provides real-time monitoring and help in decision making process. The same in [72], they exploiting the fog computing concept to provide enhancing health monitoring system. They depend on the smart gateway in analysis of ECG, extract the critical features such s P and T waves. Achieving bandwidth efficiency and decrease latency. In [73], authors provide system to control and prevent Zika virus. They used fog computing as an intermediary layer between users and the cloud server to decrease the latency and cost of communication in such high cloud-based systems. In [39] N.El-Rahsidy et al provided system for monitoring patient’s with COVID_19, it depends on combination between cloud and fog computing to improve the performance of the monitoring. 

### 3.3. Back-End System

#### Knowledge Base

This section will focus on the knowledge base layer (third layer), and its role in remote patient monitoring. A clinical decision support system (CDSS) is considered to be the brain of a healthcare system, it used to assist healthcare teams in the decision-making process [14]. The literature indicates that the use of CDSSs has an important impact on monitoring systems. CDSSs may include many functions, described as follows. (I) It provides a comprehensive health care view of the patient’s medical history. (II) Helps non-expert physicians by providing clinical guidelines, practice standards, and differential diagnoses. (III) help a patient by offering several assistive tools such as drug-schedule reminders, drug prescriptions, drug doses, drug alternatives and interactions with other drugs, devices and recommendations based on patient EHRs and knowledge bases (KB).

All these factors improve the importance of using DSS in remote monitoring systems especially in remote areas. Building decision support systems (DSSs) in health care systems gradually developed as follows. Firstly, DSSs relied on information collected from physicians, such as knowledge bases [74], but these information was insufficient alone to provide physicians with information that helped them make accurate decisions for each patient. With the widespread of RPM systems that are highly dependent on DSSs, vital signs measurements and domain experts are taking into consideration when building DSS. For example, in [75] the authors proposed a CDSS that could perform early abnormal detection using correlations between vital signs. This system used high volume cloud resources to serve many patients, reduce the hospital and predict early risks. Others [76] provided CDSS that could predict mortality based on the data of the first 24 h. In [77], the authors provided a telemonitoring system for diabetes designed to automatically evaluate patient glycemic data that uploaded according to an embedded CDSS. Despite the importance of this contribution, but it did not provide a complete view of a patient’s medical history. Therefore, a complete patient’s electronic health record (HER) consider important to build effective DSS. This importance returned to two main reasons (1) physicians may not be expert in all sub-specialties or may not have enough time for every case. (2) symptoms, information, and even vital signs supplied by patients may be interrelated in medical diagnoses, especially for elderly people suffering from more than one disease category. 

Patient EHRs considered complete source for all patient health data. It includes the patient’s medical history, vital signs, heritable diseases, radiology reports, lab tests, latest diagnoses, time, and place of their last hospital visit, etc. EHRs could provide physicians with large amounts of continuously updated information. One study that measured physician satisfaction with EHRs was presented in [50], the authors reported that physicians change to CDSS based on EHR systems, improve decision accuracy, reduce the time spent on computers, ameliorate physician fatigue and save time for patients. EHR systems have two main architectures. First, centralized EHR (local EHR) are used to communicate among healthcare systems inside hospitals, such as RIS, pharmacy, LIS, etc. Second, distributed EHRs between hospitals. These systems require considerable pre-processing and preparation work [78], since each EHR ecosystem may use different data models and/or different standards such as openEHR, HL7 RIM, ISO and CEN TC, as well as different medical terminology standards such as SNOMED CT, ICD, LOINC, and UMLS [79]. Therefore, maintaining semantic interoperability is a primary challenge in distributed EHR systems. In [80], the authors provide an EHR data model that uses object-relational data to solve problems related to dynamic design changes and sparse, time-varying or high-dimensional data. They built a model by combining both standard and generic tables to support various types of data and solve interoperability problems [78]. This model was based on the HL7 v3 RIM standard and the medical terminology SNOMED CT standard, which facilitates EHR data sharing and building distributed EHRs. A CDSS should be used as an embedded system along with an EHR and other health information system (HIS) sources (e.g., laboratories, sonograms, radiology, etc.) to support making real-time accurate decisions. In [81], authors developed a distributed CDSS that fully integrated with a distributed EHR system and distributed knowledge bases (multiple knowledge bases, i.e., heart diseases). The idea behind this study was to identify every patient with a unique number (Universal ID), which would be shared between all hospitals. When any patient visits a hospital, the system automatically supplies the patient’s profile based on the patient’s ID. Both the patient profile and current diagnoses are integrated into an XML file based on HL7 v3 to assure semantic interoperability between the patient profile and the knowledge bases in the CDSS. The CDSS will then decide and suggest any needed medication. To assure the CDSS efficiency, data mining continuously analyses various KBs and EHRs to extract the most recent knowledge and update the CDSS. 

Health level seven (HL7) is one of the main standards that support clinical delivery and exchange of electronic healthcare information, and administration of various health services. HL7 does not have specific software, but it provides healthcare organizations with specifications that make the system interoperable. Several studies have been used HL7 to resolve interoperability issues [82,83]. For example, in [84], the authors provide an EHR data model that uses object-relational data to solve problems related to dynamic design changes and sparse, time-varying, or high-dimensional data. They built a model by combining both standard and generic tables to support various types of data and solve interoperability problems [82]. This model was based on the HL7 v3 RIM standard and the medical terminology SNOMED CT standard, which facilitates EHR data sharing and building distributed EHRs. A CDSS should be used as an embedded system along with an EHR and other health information system (HIS) sources (e.g., laboratories, sonograms, radiology, etc.) to support making real-time accurate decisions. In [85], the authors developed a distributed CDSS that fully integrated with a distributed EHR system and distributed knowledge bases (multiple knowledge bases, i.e., heart diseases). The idea behind this study was to identify every patient with a unique number (Universal ID), which would be shared between all hospitals. When any patient visits a hospital, the system automatically supplies the patient’s profile based on the patient’s ID. Both the patient profile and current diagnoses are integrated into an XML file based on HL7 v3 to assure semantic interoperability between the patient profile and the knowledge bases in the CDSS. The CDSS will then decide and suggest any needed medication. To assure the CDSS efficiency, data mining continuously analyzes various KBs and EHRs to extract the most recent knowledge and update the CDSS. Table 5 shows different DSSs for various diseases. Unless HL7 v3 is developed to overcome the shortage in HL7 v2, it is criticized widely by the industry, as it is internally inconsistent, too expensive, and complex to implement. Thus, HL7 embarked on the creation of new standards known as FHIR (Fast Healthcare Interoperability Resources) [82,83,84,85,86]. FHIR belongs to the HL7 family and it took advantage of the earliest version of HL7 (HL7 v1, HL7 v2, and HL7 v3) [80]. However, it takes a different technique to maintain interoperability issues, rather than the traditional centric approach in HL7 [82]. FIHR follows the modular approach and represents the atomic healthcare data (i.e., medication, blood pressure, allergies) as independent entities called resources. All of these resources are managed (i.e., created, shared, and updated) via APSs and web services (RESTful web service) [87]. A systematic review about medical data interoperability reported that FHIR is one of the messaging standards that most satisfied the requirement of healthcare interoperability and provided several client-side features (i.e., privacy, reliability, security, flexibility, and compatibility) [88]. Therefore, FHIR is expected to have a high adoption rate. Table 6 show a comparison between HL7 v3 and HL7 FIHR.

Regarding build efficient knowledge base, fuzzy logic also has a large impact due to the special nature of medical data [95]. For example, a system was presented to predict the risks of heart diseases using fuzzy logic in [14]. they compute the frequency of specific diseases from the training dataset and then calculates a fuzzy weight used when building the CDSS. Others [96] built an assessment and evaluation expert system for heart disease patients, based on a fuzzy interference system and a fuzzy analytical hierarchy process (AHP). The AHP assigns weights to every criterion and sub-criterion and then builds a fuzzy interference system to integrate the criteria and weights in a CDSS. Fuzzy logic also helps in building case-based reasoning (CBR) systems. CBR is a paradigm that uses previously solved problems to diagnose new cases. It is based on the concept that “similar problems have similar solutions”. In [97], the author provided a case-based relational model for diabetes diagnosis and used it to build a CBR system. System based on patient EHRs, HL7 RIM, and SNOMED SCT, collect and organize data from EHRs in the form of a problem list. It contained a patient number (POMR structure), EHRs included symptoms, physical screening, lab tests, and so on. The main contribution of this system is to provide patient’s diagnosis (whether they have diabetes, the type of diabetes, the severity and complications). In [90], the authors provided a fuzzy ontology CBR to convert EHRs to a CDSS. This CBR covered symptoms, medical history, lab tests, therapy plans and treatments for all patients. Three preparation steps used to convert EHRs to a DSS (data processing, encoding, and fuzzification). First, data are pre-processed through machine learning algorithms. This step handles missing data, data normalization, and aggregation and extracts important features. The second step is unification, which converts EHRs into unified and standardized forms (e.g., HL7 RIM v3). Third, the unification step is used to enhance the representation of the patient’s data and to increase the implementation similarity.

Regrading to retrieving data from different EHR in a human-readable form, an ontology is a computer science methodology for representing knowledge as a conceptual model for a specific domain [98]. Ontologies are used to represent attributes, domain terms, concepts and the relations between them. In various systems, ontology used to store and represent knowledge in both human-readable and machine-readable forms that facilitate information retrieval tasks [93,99,100]. In [15], the authors created an ontology knowledge base for assessing diabetes patients. This ontology-based framework semantically synthesized, integrates and modeled information of patients with diabetes, including patient data, domain knowledge, and assessment parameters. Then, a service oriented CDSS was implemented within this framework to automatically adapt to the patient’s condition based on standard assessments. In [94], DSS system was developed based on an ontology to identify mental disorders (e.g., Alzheimer’s, depression, Parkinson’s and psychosis) according to a patient’s symptoms, conditions and signs. This ontology defined three classes include symptom, condition and sensor which acts as a map for making diagnoses based on signs and symptoms. Other researchers have used clinical guidelines based on ontologies to classify patient status. In [101], an ontology-based CDSS was developed for lung cancer patients. The developed ontology classified patients based on written guidelines (British Thoracic Society Guidelines) through a Lung Cancer Assistant system.

## 4. Disease-Specific Remote Patient Monitoring Systems

In this section we will discuss the patient monitoring system form different diseases perspective to present the challenges and the importance of each of them. 

### 4.1. Heart Disease Monitoring Systems

Heart problems are the most common diseases that need monitoring system [102]. This is due to heart problems are related to many illnesses and chronic diseases such as cardiac arrhythmia, chronic heart diseases. The possibility to measure and aggregate heart rate, respiratory rate, ECG, respiratory rate through wearable sensors have been discussed previously in literature. 

Some telemedicine scenario that include telecommunication center located in remote areas showed that using web applications through devices are better than specialized network protocols in emergency telemedicine system. In [103] author provide monitoring system that obtain vital signs include O2 saturation, and heart pulses. It consists of smart phone acts as hub and processor, in addition to personal area network for android. In [104] provide monitoring system that used to aggregate physiological data that related to ECG and pulmonary artery pressure (PAP), then encoding it as text. They mentioned that the continuous aggregation of medical data with the 5th generation mobile network will contribute to improve the 24*7 monitoring system. In [105] authors discuss and compare five remote monitoring system according to the monitoring method. They concluded with the efficiency of contact-based method over other contact less methods.

Unless the existence of much heart monitoring systems, there are several challenges that related to aggregate the patient’s still needing further work. For example, detect respiratory breathing abnormalities consider a challenge, as it mainly depends on breathing sound detection and the upper body produce many sounds. Therefore, it considers difficult to differentiate between them. Second, extracting heart rate and ECG that based in autonomous nervous system consider complex to implemented. 

### 4.2. Fall Detection Monitoring Systems

Falls are a primary cause of injuries, especially for elderly people [106]. In [34], the authors provided a fall detection system based on both wearable and environmental sensors. The concept was authors use a microcontroller unit (MCU) to detect falls based on prior sensor measurements. also [107], the authors proposed a safety alert system that used an accelerometer, cardio meter, and smartphone. They constructed a network between these sensors via Bluetooth. When any abnormal sensor readings occurred, it automatically informed health care professionals and the family and identified the patient and their location

In [108], authors provide (IF-Then rules) for fall detection that depend on embedded sensor, the sensor used to detect falling without any database connection. [103] provide fall detection monitoring system based on android system, in [106] author provide fall detection system that based IoT. This system depends on echo device, webcam, and speaker. All these devices connected by hub (raspberry pi). When any fall detected, the device connected to the hub and the speaker speak to the patient quickly. The main challenges with fall detection system is differentiate the fall from the daily activities of the patient, therefor; the false alarms are common on these kind of monitoring systems. Another challenge with this system is the lack of data to improve the research in this domain. 

### 4.3. Mental Health Systems

Mental health systems are usually used to help patients with memory impairment like a patient with Alzheimer’s. In [109] provide literature review for all technologies of mental health monitoring systems. In [110], authors proposed a system that reminded patients with medication dosages at pre-determined times, monitored patient compliance using a Microsoft Kinect and embedded sensors. Others [111], used wristband sensors to enable a medication-compliance monitoring system, the idea behind this system was to use a machine-learning algorithm to build and train a classifier to track wrist motion and interference to the schedule for medication adherence. Other medication adherence monitoring and reminder systems were provided in [32,33] in [112] authors provide monitoring system for patients with mental bipolarity. It based on gathering speech and other features during patient’s activities, in addition to sleep and medication activities using nervous sensor system. In [113] provide system for monitoring patients with Parkinson’s that have passive and active monitoring system. Other for Alzheimer’s [114]. The main challenge with this system is the complexity of designing, this is because mental health diseases may associate with other diseases, which demands continuous monitoring for vital signs, nervous and psychiatric response [115]. 

### 4.4. Diabetes Monitoring System

A recent research in diabetes monitoring systems aim to compare the continuous monitoring system with the traditional monitoring in terms of acceptability, efficient and accuracy. In [115,116] provide monitoring system for diabetes that based on automatic feedback massage, it record patient blood glucose level, food intake and blood pressure, in addition to patient physical activity, all of them used to make chatting with patient and manage the glucose prescription. In [117] propose complete framework for diabetes patients, it integrate with CDSS capabilities. They depend on FASTO ontology to construct semantic CDSS that could manage patient health status regardless of the quality of knowledge.

### 4.5. Vital Sign Monitoring and Health Assessment Systems

Real-time patient monitoring and continuous assessment systems are a particularly useful tool that helps in analyzing patients’ health status. It used to monitor patient’s activities, predict early risks, and so on. In our study period, several studies were concerned with providing PM systems. For example, [29] authors proposed a monitoring system for heart diseases, monitor a patient’s vital signs (SpO2, ECG, blood pressure) and then transmit the data according to the patient’s health status through four main transmission modes (continuous transmission for all data, continuous transmission at special times, event-triggered transmission and transmission on demand). Other studies [30,118,119] have proposed similar ideas and provided health monitoring systems for elderly people that support continuous follow-up and risk prediction. In [31], a health assessment system was provided for patients with dementia, authors hypothesized that cognitive health could be estimated by continuously monitoring patients’ daily activities. Their system applied signal processing to data aggregated from wearable sensors (i.e., accelerometer and electrodermal activity (EDA)). Machine learning techniques are used to evaluate cognitive health status and its correlation with patient daily activities. In [120], authors provided a health cognitive assessment system according to activity behavior (CAAB). System extracted features that model activity performance, then use supervised and unsupervised machine learning algorithms to evaluate the patient’s cognitive health status. The same idea was discussed in [121], the authors built a classification model that classified patients into one of three groups based on their cognitive health status.

### 4.6. Other Diseases Monitoring Systems

In [122], the authors proposed mobile PMs for chronic patients that need continuous and long-term monitoring. This system aims to decrease the need for hospital visits as well as nursing. The same in, authors provide both passive and active monitoring systems for chronic disease patients [123]. In [124] authors provide the DeStress mobile monitoring system that is used to collect, analyze, and share stress data between physicians to provide real-time interactions in response to critical events and provide specialized feedback for each patient. In [125], the authors provide indoor PMs, they developed a mechanism to locate patients inside their activities and detect any changes in their behaviors that could be used to detect any critical health events. 

## 5. The Role of Artificial Intelligence in RPMs

In recent years, there has been an increased emphasis on using Artificial Intelligence (AI) to solve complex problems in different domains. In the healthcare sector, AI contributes to changing the way of healthcare delivery in different healthcare settings including clinics, hospitals, etc. The main objective of AI is to build systems that can simulate human thinking, using a collection of technologies to perform various healthcare tasks with a better than human performance, and utilizing AI techniques in analyzing patient’s data and predicting future events, building an intelligent interface that interacts with the patient and increases his/her engagement with the therapy plan. Then, using this information to increase patient independence and awareness. For example, in [126], Sturiale et al. presented a large survey on 5800 colorectal patients. They analyzed the correlation between using the internet and social media for work and the time for consultations. They reported that patients that used the internet made the first consultation after six months of symptoms appearance (*p* < 0.0001), and likely to know about their diseases before diagnosis. This study confirmed the effective role of the internet and social media in increasing patient participation in therapy plans. In this section, we concentrate on the most important AI technologies related to PMs, spotlight their potential to automate various aspects, and overcome barriers in building PMs. It is summarized in the following points. 

### 5.1. Rule-Based Systems (Expert Systems)

Most PMs involve the observation and the examination of patients through prognosis and diagnosis systems. AI is used for building these medical tasks, it compares the medical diagnosis with the intelligent agent system, where the medical experts consult the intelligent agent and patient data and the diagnosis considers the input and the output. Several AI techniques could be used to assist medical experts on this side; one way is to use what is known as expert systems. Expert systems based on several “IF-Then” rules map between inputs and outputs. These rules are built with the help of medical experts who have deep experience in a specific domain [127]. The success of these systems depends on the representation of knowledge in the form of logical rules. However, if the number of rules is large, they may conflict with each other and result in a difficult and time-consuming system. Consequently, it is slowly being replaced with more sophisticated approaches based on data mining and machine learning techniques.

### 5.2. Machine Learning Techniques

Machine learning (ML) is a broad AI technique used to build models that could learn from data. As patients increase, ML and AI help in providing healthcare systems that could deliver care more appropriately and efficiently. In RPMs, ML is used to build models that help predict risks and provide diagnosis and treatment based on medical data. The following sub-section serves ML applications and techniques used in RPMs.

(1) Supervised and non-supervised algorithms: Several types of supervised machine learning algorithms are used in RPMs, analyzing medical data in order to predict patient future events. For example, El-Rashidy et al. [80] used supervised ML algorithms (i.e., rule-based classifier, non-linear classifier, instance-based classifier, tree-based classifier, etc.) to analyze patient’s medical records and predict mortality among them. Each classifier used a different learning algorithm to build a model that best fit between input and output with a good generalization capability. Shamer et al. [128] developed a quality assessment model that was used to predict readmission, several ML algorithms were integrated to build an ensemble model to make predictions. The same model was used for predicting complications in ICU units [125], cardiovascular [129,130] Diabetes [131,132,133,134], sepsis [135,136], and COVID-19 [136,137,138]. ML is also used to provide timely medical services to patients. One such example is called Home Smart Health (HSH) [139]. HSH used a body sensor network and a personal sensor network for building a smart environment that has the capability to meet patient’s needs. ML (supervised and non-supervised) is used to analyze patients’ data (sensor data) to understand patient behavior and provide specific services for each patient.
(2) Reinforcement learning (RL): ML models that learn by the trial-and-error concept, the learning process is repeated until the optimal solution is reached. RL is used in various monitoring systems. For example, Nuayto et al. [140] built RPMs for continuous monitoring of bio signs through a heterogeneous sensor transceiver. The proposed architecture used reinforcement learning (constrained Markov decision process (CMDP)) to minimize cost while maintaining the optimal quality of service (QoS). Wipawee et al. [141] used Q-learning (reinforcement learning) to provide a monitoring system. They used a distributed routing mechanism to route information to the nearest sink. Others use reinforcement learning to find the optimal treatment for a patient with anemia.
(3) Deep learning (DL): This is a new area of ML that simulates the human thinking process. DL provides healthcare applications the ability to analyze huge data at exceptional speed with promising accuracy. For example, El-Sappagh et al. [115] used the DL model to predict patients with Alzheimer’s based on patient vital signs and X-ray images. Other common applications use DL models to specify the most critical features in patients’ imaging data, it is considered a promising solution in oncology image analysis. DL also has an increasing impact on natural language processing (NLP) [142]. In RPMs, NLP contributes to understanding the clinical notes on patients to provide efficient monitoring, transcribe interactions from patients, and provide conversational AI supportive tools such as chatbots [143]. For more details, [21,141,144] provide comprehensive surveys about using ML in RPMs.

### 5.3. Human-Computer Interaction

Human-computer interaction (HCI) is concerned with building intelligent interfaces that could communicate with patients. It studies the relationship to build two-way, unobstructed communication between patient and system [145]. Along with the development of monitoring systems, one of the main goals is to carry out close monitoring of patients’ physiological features and meeting the needs of each patient according to his/her status, which is the main objective of HCI. Mustfa et al. [146] provide a comprehensive survey about the importance of HCI in the healthcare sector. Good HCI design of RPMs facilitates the effective monitoring of patient physiological parameters, assists patient to the cure, and interacts effectively and quickly. For example, Liu et al. [147] provide the design of an HCI monitoring system for the ICU. This system aims to reduce the workload by developing a system that could adaptively respond to patient’s needs. 

### 5.4. Physical and Processing Robots 

The shortage of healthcare expertise has been a big challenge in the last years, especially in the developed countries. Hospitals are overcrowded with critical cases that need continuous monitoring. With stressed-out medical experts, the probability of medical errors may be high frequency. In the healthcare sector, robots help by providing safer and less costly medical procedures. Recently, robots have become more intelligent, as various AI techniques are being embedded in their operating systems. Formerly robots could work as medical assistants that continuously monitor patient’s vital signs and provide alerts to the nurse if the patient needs human presence, they can also assist in conducting critical surgeries [148]. With the growing spread of the coronavirus (COVID-19), robots participate in various applications including a telemedicine robot that helps doctors in monitoring the physiological parameter of large-scale patients remotely. Nursing robots [149], which were developed to assist physicians in the same manner as humans, provide care to various patients while limiting the spread of infection. Radiologist robots [150] can take several X-ray images and permit a physician to see the 3D images in real-time through the robot rather than the patient. There are also rehabilitation robots [127] and ambulance robots [151].

Unless we believe in the ability of AI to reshape the future of the healthcare sector through various technologies and applications, the greatest challenge that AI faces is not to develop useful applications but to ensure their adaptability to patients’ clinical practice. AI applications should integrate with patient’s EHR systems, standardized in a way that permits the application to interact with the other applications in the same domain. In the future, we expect that AI will not replace physicians and nurses but will save their effort to provide more care for patients. 

## 6. Case Study: Chronic Diseases Monitoring System

**Figure 5:**
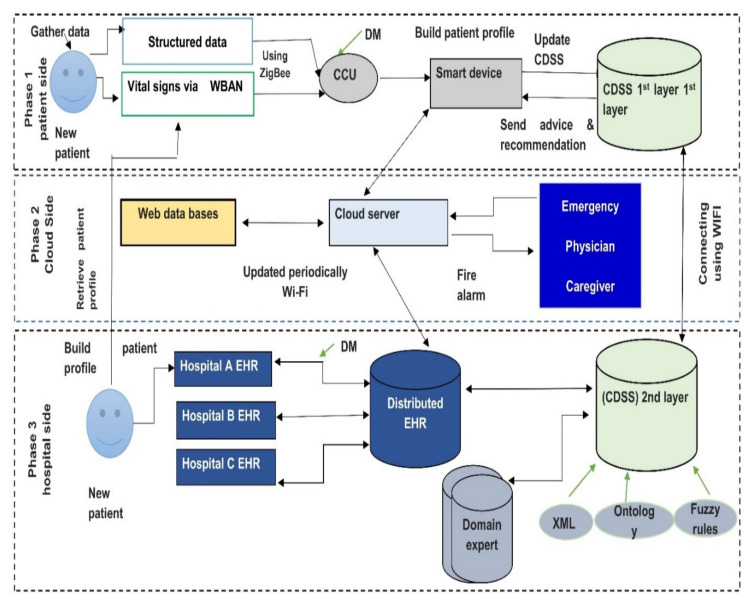
Case study for monitoring system for chronic disease patients.

In this section, we discuss a case study for RPMs to provide a framework that utilizes various technologies to handle some challenges of the literature. In this case study, we propose a framework that could be used to monitor all chronic diseases. Patients with chronic diseases need continuous monitoring for all vital signs to avoid patient deterioration. Most of the monitoring systems focus only on monitoring specific vital signs. For example, most diabetes monitoring focuses on blood glucose as it is the most appropriate way when diagnosing diabetes. Despite the importance of blood tests in diabetes diagnosis [16], the other vital signs should be considered in monitoring diabetics, as when glucose level increases, blood vessels negatively affect the kidney, heart, vision, etc. Therefore, it is important to measure other factors besides the glucose level such as blood pressure, body temperature, conscious level, respiratory rate, etc. to avoid diabetes complications. In this case study, we try to overcome some challenges that were previously mentioned in this study. The execution of the proposed monitoring system can be summarized in the following steps, see Figure 5.

(1) Lightweight biosensors are attached to a patient body. They continuously monitors patient vital signs like glucose level, vision level, fatigue level (EEG), activity level, blood pressure, body temperature, etc. Then all vital signs are gathered and sent to the central control unit. Note that ZigBee is used to deliver vital signs from sensors to central devices. If mobile applications notice that there is no patient record, the system will send a message to the patient via text or call to check the sensor or batteries. In case of no response after a short time system will automatically call the caregiver to check the patient’s state.
(2) Social media patient’s activities (Facebook comments and tweets) are also tracked continuously and analyzed using components for handling unstructured data. All gathered raw data are then transported to the central control unit (CCU). In some cases, a smartphone may be used as a central control unit.
(3) Our proposed framework provides two monitoring modes, the online and offline monitoring systems. The offline mode runs via the first layer CDSS that is installed on a personal server (discussed in the next step), and online via a cloud server (discussed in step 4), distributed her, and second layer CDSS. In the personal server, each patient transmits his/her vital signs, then all patient’s data are transmitted to the cloud hospital server.
(4) In case the internet connection is interrupted or unplugged, the system will not work properly, and the patient will not be able to connect with the system. To overcome this challenge, a light CDSS was added to the patient side to monitor the patient until the internet problem was fixed. The CDSS’s first layer helps patients with advice and recommendations based on the patient profile (i.e., EHR) and a small knowledge base. The knowledge base will continually update by discovering and extracting knowledge from the EHR. CDSS in the first layer resolves the human-computer interaction issues and provides a simple and user-friendly GUI that does not require experience in dealing with computers or smart apps.
(5) Periodically, patient data is transmitted to a stand-alone device where a wireless area network is created between it and another system component (Caregiver provider, family, emergency system), which permits them to access and check the patient status and retrieve patient information during monitoring system. In case the system detects abnormal signs, it will fire the alarm and send an alarm message to the network. Note that Wi-Fi IEEE 802 is used to transmit data between CCU, cloud server, and the CDSS second layer.

The CDSS service provider’s side provides comprehensive and complicated decision support based on the patient’s entire historical data (second layer CDSS). The knowledge base for this CDSS updates periodically with the most recent updates in each cloud-EHR for each hospital. This CDSS system utilizes the semantics of ontology and fuzzy logic to optimize the resulting decisions. It supports the prediction of the patient’s future conditions and suggests preventive actions by mining the temporal data collected. Finally, if the two CDSS systems fail, the healthcare personnel are contacted. The first and second CDSSs act as a virtual doctor. This framework will be implemented in our next paper.

## 7. Study Results

In Table 7, we make a comparison of the similarities and differences and classify patient monitoring systems according to six criteria (use of data mining technique, collecting vital signs with WBAN sensors, sharing data in cloud servers for storage process and sharing, using ontology and semantic interoperability in diagnosis and EHR environment, CDSS as real-time advice). The symbol (🗸) indicates that the research paper uses the checked technology and the opposite is indicated by the symbol (🗴).

Currently, many health monitoring projects and applications have been initiated that use different architectures. Health monitoring systems are heterogeneous and have been developed for various diseases and disabilities. Table 8 shows a list of remote patient monitoring projects and applications associated with WBAN-based health monitoring systems.

### Challenges and Future Directions

According to the previous literature surveys and despite the benefits of RPM systems, current systems have many limitations and challenges that affect the effectiveness of diagnosis and treatment and generally affect the appetite for using e-health systems. Handling these challenges will improve system capabilities and increase patient stratification and acceptance. In this subsection, we summarize the common challenges and discuss the possible solutions that could help to reduce their impact in the future.

- Not all smart devices support the automatic transmission of patient data to the cloud or the fog nodes without patient intervention. Therefore, a new generation of mobiles should work on providing the automatic and accurate transfer of data [166]. For example, in 2016 Android worked on improving the sampling rate constraints and permitting third-party applications to sample from various sensors.
- The accuracy of sensing devices (i.e., sensors) has still not reached a stable state; therefore, various challenges include working on enhancing signal processing and transmission. For example, Kim et al. [167] introduced a group of analog-front-end solutions that address the tradeoff between the quality of transmission and power consumption.
- The RPM systems are developed to solve the problem of patient monitoring regardless of time and place. Therefore, the design of WSN should maintain the mobility, transmission rate, data rate, and network coverage issues [168]. For example, building monitoring systems that utilize both fog computing and cloud computing may provide various capabilities such as mobility, low latency, and low bandwidth consumption.
- Managing and integrating the massive data extracted during patient monitoring are considered a daunting task. To take full advantage of the extracted data, various data mining and knowledge extraction tools should be developed to have deep insights into these data to improve knowledge outcomes and decrease costs [76].
- The internet is considered the primary medium for data transmission in any RPMs. This raises the need for ironclad privacy and security protocols to protect data from different attacks such as data eavesdropping modification and impersonations. The problem worsens due to the fact that most wireless body area network devices used in patient monitoring are limited in memory, processing, and energy capabilities [169]. Therefore, it is considered impossible to provide full monitoring systems based on them. Accordingly, privacy and security issues need additional work, to provide an acceptable solution in the different layers of monitoring [170,171]. A comprehensive survey of security and privacy in patient monitoring can be found in [172].
- Encryption could be used to prevent data eavesdropping. Therefore, working on symmetric and asymmetric key encryption algorithms could help to provide a high level of security for patient’s data [173].
- Managing large networks is also a complex challenge. Therefore, working on developing role-based access control systems may help in reducing the complexity in administration, especially with large healthcare systems.
- Monitoring systems could be used for a small number of patients in clinics or may be scaled up to be used by a large number of users in hospitals. This results in the rapid growth of demands for physicians as well as healthcare organizations. Accordingly, RPMs should be scalable in terms of applications, networks, and services [8].
- RPM systems are very time-sensitive and require the guarantee of several QoS criteria such as maintainability, reliability, and availability. This is due to the fact that such systems put patient’s lives in danger in critical health problems [174].
- The power consumption of WBAN sensors is a big challenge for RPMs. Usually, the capacity of batteries is consumed in sensing, processing, and transmitting of data, so that it requires frequent recharging. It may be considered the weakest point in RPMs as frequent charging for batteries is considered a big burden for patients. Therefore, the optimization of power consumption is considered one of the main points in various studies. Some studies working on improving the current protocols such as Zigbee and Bluetooth are [11,132,175]. Others work on extending the lifetime of the sensor battery by utilizing medium access control (MAC) protocols with low power consumption [176].
- Providing continuous monitoring in the healthcare sector requires the use of various sensors that are mostly manufactured by different manufacturers. The lack of standardization techniques hinders the ability of devices to communicate and transmit data among them effectively. Therefore, working on standards and data integration protocols is considered a pressing need to provide data and device interoperability. From the application side, some monitoring applications require approval for use from some bodies such as the FDA. To overcome this delay, participants must come up with medical guidelines that work on speeding up the deployment of medical applications.
- The development of complete RPMs that allow patients to integrate with various hardware and software service providers and different sources of data (heterogeneous sources with different standards and formats) is a challenge that needs to be addressed in future studies.
- The patient’s EHR system may include various components such as laboratory systems, hospital information systems, etc. Each component may have different standards (i.e., HL7, OpenEHR, and ISO/IEEE) and different terminologies (i.e., LONIC, SNOMED CT, and CPT4). Therefore, working on a unified standard is essential to maintaining syntax and semantic interoperability [177].
- CDSS should work based on a patient’s EHR data, in addition to vital signs data sensed from wearable sensors. Therefore, CDSSs should provide specific services based on each patient’s data. On the other hand, CDSSs interfaces should maintain a brain-computer interface (BCI) and human-computer interaction (HCI) in order to support the dynamic creation of an application interface according to patient’s moods [178].
- Based on the surveyed literature presented in this paper, we could not categorize whether the existing RPM solutions are easily compatible with security and privacy legislation. Nonetheless, as healthcare solutions undergo a digital transformation, the paradigm needs to be implemented with the compliance of different legislative frameworks such as the general data protection regulation (GDPR) and network and information security (NIS) directive (NISD) requirements [82]. While the GDPR is a privacy directive that instructs how organizations should handle personal data, the NISD emphasizes strengthening organizations’ security capability from the service infrastructure viewpoints. The work in [83] identified a set of different measures that can be integrated with m-health systems to adopt GDPR-compliant security and privacy schemes. Recently, the work in [179] provides a case study of the “WELCOME” research project, an integrated system for chronic patients’ monitoring, diagnosis, detection, and treatment. In the study, the authors propose a framework for the security and privacy of m-health applications adhering to the GDPR guidelines. Policy enforcement is necessary to monitor and guarantee that the digital information systems strictly follow specific policies in dealing with medical information.
- The advanced message queuing protocol (AMQP) and message queuing telemetry transport (MQTT) are the two most common data transfer protocols used to exchange data between IoT systems and edge or cloud servers. Although both of these schemes are non-healthcare-specific protocols, they can be integrated with HL7, which is here and now the most widely adopted data interaction standard in medical applications [180]. In MQTT, a broker receives messages from the publishers then routes the messages to the respective subscribers. While AMQP provides similar functionality as MQTT, it also facilitates queues in the broker to store the message when the consumer does not access the messages. Large organizations that include many IoT devices require a higher level of data integrity. Therein, both AMQP and MQTT can simultaneously be deployed for different clusters and regions [181,182]. As such, the coexistence of AMQP and MQTT protocols is conceivable in HL7-facilitated organizations. However, to determine the suitability for the HL7 framework, more research on lightweight publish-subscribe network protocols is required from practical and implementation contexts.

Despite having overcome many previous challenges, one of the main challenges still facing RPMs is how to convince chronic disease patients, particularly elderly people and their relatives, to alter the way they look at RPMs and ensure peoples’ trust when using them. In a report conducted by the Deloitte Center for Healthcare, RPMs not only face technological challenges from sensors and protocols but also social, cultural, and educational challenges from patients and their convictions. Most of the challenges in current RPMs revolve around the inability to access all patient data in real-time, and this deficiency prevents physicians from gaining a continuous and complete view of the status and condition of their patients. These drawbacks make RPMs less effective in the diagnosis, monitoring, and treatment of chronic diseases.

## 8. Conclusions

In this paper, we tried to provide a comprehensive survey about RPMs. RPMs are usually built on three layers. The first layer is concerned with data acquisition, it is used to gather data using invasive or non-invasive sensors. All patient measurements are transmitted to the second layer using various transmission protocols. The second layer consists of cloud and web servers that receive, process, and store data for further use. The third layer is the back-end layer. It uses all the patient data to develop a complete patient EHR, it may also include CDSS, KB, CPG, CBR, etc. that are based on patient EHR and medical knowledge, etc. This layer helps in early detection and intervention. Each layer faces several complexities and challenges that hamper building effective real-time RPMs. In this review, we tried to highlight challenges related to each layer. To achieve this goal, we surveyed 56 papers related to RPMSs for different diseases according to the current technology. We expect that in the near future, continuous monitoring could be done using cheap sensors, all recording patient’s data transmitted into a complete EHR. This study may help researchers interested in PMs to better understand the current state of evidence that is available in the literature and assist in the planning of future research to address challenges and limitations, which may address the gap identified in this review. In the future, we intend to conduct another study that covers other issues such as privacy and security issues, hardware and sensors, and other related points.

As with the majority of studies, the design of the current study is subject to some limitations. The current study focused on RPMs for chronic disease, we tried to address it from different perspectives. However, some limitations should be noted. First, we mainly focused on RPMs from the software side, while hardware (i.e., type of sensors, servers, and network components) is out of our interest in this study. Second, privacy and security issues in PMs spans the overall lifecycle of the monitoring systems, from wireless sensors and devices, cloud systems, to backend systems are discussed briefly. Third, we did not include the different ways of data transmission in RPMs, and how the transmission could be varying according to the data type (i.e., text, image, video, signal, etc.). Fourth, issues related to the physical positioning of patient data in terms of legal and governmental regulations should also be discussed. As some countries prevent storing and processing patient’s data outside hospitals. Overall, although limitations are acknowledged from a general viewpoint, our contributions in this study will ideally motivate others to resolve the existing problems in the field because the highlighted limitations are not contradictory to the primary objectives.

## Figures and Tables

**Table 1:** Keywords used to obtain eligible articles.

	Key Words	Databases					Total Publication Identified
#		Science Direct	IEEE	Springer	Scince.gov	PubMed	
1	Remote patient monitoring	326	619	506	699	800	2950
2	Remote patient monitoring AND clinical decision support system	4	160	118	267	29	578
3	Remote patient monitoring AND ontology	16	18	23	237	44	338
4	Remote Patient monitoring AND data mining	24	46	42	84	23	219
5	Remote patient monitoring AND wireless body Area network	16	15	30	102	10	173
6	Remote patient monitoring AND ontology AND (cloud computing OR Fog computing)	8	7	85	42	2	144
7	Remote patient monitoring AND ontology AND cloud computing and wireless body area network AND clinical decision support system	1	0	5	2	3	11
Total		395	865	809	1433	911	4413

**Table 2:** Transmission protocols.

Power Requirement	Frequency	Coverage	Transmission Protocol
Very Low	2.4 GHz	70–100 m	Zigbee
Medium	1 MHZ	10 M	Bluetooth
High	2.4 GHZ	100 M	Wi-Fi
Low		10 KM	LoRa

**Table 3:** Description of selected articles.

#	Diseases	Collected Data	Sensor	Transmission Protocol
[26]	Heart diseases	ECG	ECG monitor node	Wi-Fi (HTTP, MQTT)
[27]	Heart diseases	ECG	ECG fabric sensor embedded on the patient’s chair	Bluetooth
[28]	Pain assessment	Facial expression (sEMG)	Wearable sensor with a bio-sensing facial mask	Wi-Fi
[29]	Heart diseases	Spo2, blood pressure, ECG		Wi-Fi
[30]	Heart diseases	ECG	Wearable smart clothing	Bluetooth
[31]	Dementia	Changes in behaviors and Functional health	Electrodermal Activity (EDA), Photoplenthys (PPG), Accelerometer (ACC)	Wi-Fi
[32]	Chronic diseases	Monitor medication adherence	Smart home sensors	Wi-Fi
[33]	Chronic diseases	Monitor medication adherence	Wristband wearable sensor	Bluetooth
[34]	Fall detection	Monitor mentions and predict falls	Accelerometer, Cardiotachometer	ZigBee
[19]	Heart diseases	Spo2, HR	Wireless pulse oximeter	Wi-Fi
[35]	Hypertension	Blood pressure	Electronic blood pressure measurement	Bluetooth

**Table 4:** Difference between cloud computing and fog computing.

Factor	Cloud Computing	Fog Computing
Delaying	High	Low
Mobility ability	Limited	Supported
Geo-distribution	Centralized	Distributed
Bandwidth consumption	High	Low
Storage capabilities	Strong	Weak
Power consumption	High	Low
Location identification	Partially supported	Fully supported
Number of servers	Few	Large
Real-time interaction	Supported	Supported
security	Undefined	Defined
Service location	With the Internet	At the edge of the local network

**Table 5:** Description of CDSS papers.

Performance	Methods	Data Collection	Diseases	#
99.30%	Ontology, interoperability, CDSS	115,477 records collected from of 36,162 type 2 diabetic patients	Chronic diseases	[15]
-	Ontology, sensors	Ontology tested on “SPARQL” Query	Cardiovascular	[2]
87%	Fuzzy logic, ontology reasoning	The system evaluated in Taichung Hospital in central Taiwan	Diabetes	[89]
97.67%	Fuzzy ontology CBR	60 real cases from Mansoura university hospitals	Diabetes	[90]
	Machine learning	90 patients with gestational diabetes	Diabetes	[77]
92%	Case base finding	323 real cases	COPD diseases	[91]
90–95%	Machine learning (24 classifier combination)	85 patients	Real time monitoring	[92]
89%	Machine learning, ontology	Real-time patient data form Biosensors	Mental disorders	[93]
-	Ontology-driven	English lung cancer dataset (LUCADA), approximate (115,000) patient recode	Lung cancer	[94]

**Table 6:** Comparison between HL7 v3 and HL7 FIHR [84].

Factor	HL7 v3	HL7 FIHR
Year of initiation	1997	2011
Development Methodology	Top-down	Incremental
Semantic ontology	Yes	Yes
Architecture	Massages	RESTful web services
Tooling required	Yes, just compiler	No
Industry support	Weak	Yes
Adoption degree	Low	Expected to be high
Industry support	Weak	n/a
Character support?	Yes (conceptually)	Yes (UTF8)
Massage format support	Realm	Global standard

**Table 7:** Comparison between RPM Papers.

#	Diseases	DM	IoT	WBAN	Cloud	Ontology	Interoperability	CDSS
[15]	Chronic diseases	🗸	🗴	🗴	🗴	🗸	🗸	🗸
[2]	Cardiovascular	🗸	🗴	🗴	🗴	🗸	🗴	🗴
[26]	Heart diseases	🗴	🗸	🗴	🗸	🗴	🗴	🗴
[152]	Ubiquitous monitoring system	🗸	🗸	🗸	🗸	🗴	🗸	🗸
[28]	Pain assessment	🗴	🗸	🗸	🗴	🗴	🗴	🗴
[29]	Heart diseases	🗴	🗸	🗸	🗸	🗴	🗴	🗴
[40]	Knees rehabilitation	🗴	🗴	🗸	🗸	🗴	🗴	🗴
[153]	Vital signs gathering and processing	🗸	🗴	🗴	🗸	🗴	🗴	🗴
[46]	Chronic diseases	🗴	🗴	🗸	🗸	🗴	🗸	🗸
[47]	Hypertension	🗸	🗴	🗸	🗸	🗴	🗴	🗸
[57]	Tracking daily activities	🗴	🗴	🗸	🗸	🗴	🗴	🗴
[61]	EXP carried on healthy volunteers	🗴	🗸	🗸	🗸	🗴	🗴	🗴
[92]	Context aware monitoring	🗴	🗸	🗸	🗸	🗴	🗴	🗴
[77]	Diabetes and Diet monitoring	🗴	🗸	🗴	🗴	🗴	🗴	🗸
[96]	Heart diseases	🗴	🗴	🗴	🗴	🗴	🗴	🗸
[97]	Diabetes	🗴	🗴	🗴	🗴	🗸	🗸	🗸
[90]	Diabetes	🗸	🗴	🗴	🗸	🗸	🗸	🗸
[93]	Mental disorder	🗸	🗴	🗴	🗴	🗸	🗴	🗸
[154]	Chronic diseases	🗸	🗴	🗴	🗴	🗸	🗴	🗸
[155]	Monitor patients with depression	🗸	🗴	🗸	🗸	🗴	🗸	🗸
[131]	Cardiovascular diseases	🗸	🗴	🗸	🗴	🗴	🗸	🗸
[156]	Hypertension, hypotension	🗸	🗸	🗸	🗸	🗴	🗴	🗸
[157]	Diabetes	🗸	🗴	🗸	🗴	🗴	🗴	🗴
[158]	Heart diseases	🗸	🗴	🗸	🗸	🗴	🗴	🗴
[159]	Knee arthroplasty	🗸	🗴	🗸	🗸	🗴	🗴	🗴
[160]	Elderly	🗸	🗴	🗸	🗸	🗴	🗴	🗸
[161]	Diabetes	🗸	🗴	🗸	🗴	🗴	🗴	🗴
[162]	Parkinson’s disease	🗸	🗴	🗸	🗴	🗴	🗴	🗸
[106]	Fall detection	🗸	🗴	🗸	🗴	🗴	🗴	🗸
[117]	Diabetes	🗸	🗴	🗸	🗴	🗸	🗸	🗸
[116]	Alzheimer’s	🗴	🗴	🗴	🗴	🗴	🗸	🗸

**Table 8:** Remote patient monitoring projects and applications.

System	Year	Description	Accuracy
Help4Moodproject [155]	2014	Health care system designed to help people with depression to return to their normal life, the system consists of three main component, (1) personal server to monitor patient behavior such as sleep activity, (2) interactive agent that interact and collect information from the user through questionnaire (3) DSS that analyze patient collected	
SHARE [47]	2015	RPM system based on cloud computing, system propose proactive monitoring based on data mining functions, system combine CDSS that designed to respectively train and test the new data and adapt the system to predict vascular for whole the next year.	67%
VISIGNET [46]	2014	RPM system for chronic diseases, system monitor vital signs (Body temperature, blood pressure, and heart rate) then send it to the cloud, the system permits patients and physicians to watch health data. In addition to that, they also provide visualization watch that classifies each vital sign according to special criteria.	95%
M4CVD [131]	2015	RPM for monitoring cardiovascular diseases that use wearable sensors to collect vital signs (Blood pressure, galvanic skin response (GSR) that indicate stress level, Electrocardiogram (ECG)), the system proposes a contribution to optimizing system effectiveness by analyzing data in the local device (smartphone), it was done using a machine learning algorithm (SVM) that classify patient data and extract the clinical features to determine patient condition “continued risk” or “no longer risk”.	90.5%
WANDA [163]	2019	A monitoring system for Cognitive heart failure (CHF) patients, it consists of three tiers (first layer: biosensors for monitoring patient data. Second layer: a web server that store and maintain data integrity layer between different healthcare providers, this layer also analyze data and sends an alert message via text message or emails. Third layer: back-end server backup and recovery layer by making an offline backup)	----
Health@Home project [164]	2016	A remote monitoring system for cardiovascular diseases, the system has client/server architecture. Client-side: located at the patient side, consists of a set of biomedical sensors that measure patients of vital signs (ECG, SPO2, Chest impedance, respiration, blood pressure), then the measured sensors send through the gateway to the server-side. ADSL or mobile broadband (UTMS/GSM) used to transmit data. Server Side: installed at health service facilities, process and analyze data from gateway using the expert system, and make it available for consultation, and finally patient record in the patient information system (HIS). The system also provides an alarm system that sent by a short message to the physician, patient, and relatives.	
Nevonprojects [165]		The system is used to track patient health status via two main sensors (temperature sensor and blood pressure sensor). Sensors are connected to a microcontroller that tracks patient status.	

## Abbreviations

AHP	Analytical hierarchy process
CBR	Case-based reasoning
CC	Cloud computing
CDSS	Clinical decision support system
CHMS	Cloud health monitoring system
COPD	Chronic obstructive pulmonary diseases
ECG	Electrocardiogram
EEG	Electroencephalogram
EHR	Electronic health record
EMG	Electromyogram
HL7	Health Level Seven
IoT	Internet of Things
KB	Knowledge bases
MAC	Medium Access Control
PMS	Patient monitoring systems
PRISMA	Preferred Reporting Items for Systematic Reviews and Meta-Analyses
QoS	Quality of service
SCI	Spinal cord injury
SNOMED-CD	Systematized nomenclature of medicine-clinical terms
TDMA	Flexible time division multiple access
Term	Abbreviation
UMLS	Unified Medical Language System
WBAN	Wireless body area network
XML	Extensible Markup Language
